# Dectin-1-Syk-CARD9 Signaling Pathway in TB Immunity

**DOI:** 10.3389/fimmu.2018.00225

**Published:** 2018-02-13

**Authors:** Matthew Wagener, J. Claire Hoving, Hlumani Ndlovu, Mohlopheni J. Marakalala

**Affiliations:** ^1^Division of Immunology, Department of Pathology, Institute of Infectious Disease and Molecular Medicine, University of Cape Town, Cape Town, South Africa; ^2^Department of Integrative Biomedical Sciences, Faculty of Health Sciences, University of Cape Town, Cape Town, South Africa

**Keywords:** tuberculosis, *Mycobacterium tuberculosis*, dectin-1, C-type lectin receptors, innate immunity, TB immunity, pattern-recognition receptors, pathogen-associated molecular patterns

## Abstract

One of the first steps toward mounting an effective immune response to *Mycobacterium tuberculosis* (Mtb) is recognition of the pathogen through pattern-recognition receptors (PRRs) expressed by innate immune cells. Activation of the PRR Dectin-1 by an unknown mycobacterial ligand triggers an intracellular signaling cascade involving numerous proteins, including spleen tyrosine kinase, protein kinase C-delta, and caspase recruitment domain family member 9, some of which have been shown to influence host immune response to TB infection. Here, we review the role of Dectin-1 signaling pathway in anti-mycobacterial immunity and discuss its contribution in the control of Mtb infection, and potential applications in TB vaccine adjuvanticity.

## Introduction

A critical function of the innate immune system is differentiating between cells or components which are “self,” and those of pathogenic microorganisms (1). Innate immune cells express pattern-recognition receptors (PRRs) which recognize evolutionarily conserved microbial molecules, known as pathogen-associated molecular patterns (PAMPs) (2). Recognition *via* PRRs such as C-type lectin receptors (CLRs), NOD-like receptors, and toll-like receptors (TLRs) enable these cells to initiate responses to a broad range of potential pathogens.

When an innate immune cell, such as an alveolar macrophage, recognizes *Mycobacterium tuberculosis* (Mtb), a unique complement of PRRs will be activated. This triggers a signaling cascade within the innate cell, which results in the expression of immune modulators tailored to that pathogen. These immune modulators may trigger local inflammatory responses and provide the costimulation required for the activation and proliferation of adaptive immune cells, such as CD4^+^ T cells (2).

Understanding the role that PRR signaling plays in immunity to mycobacteria has been a recent research focus. Recent studies have shown that some receptors that activate the spleen tyrosine kinase (Syk)/caspase recruitment domain family member 9 (CARD9) signaling pathway may contribute to anti-mycobacterial defense. Several CLRs that recognize mycobacteria are known to utilize this pathway, including Dectin-1, Dectin-2, Mincle, and Clecsf8 (3, 4). These receptors are briefly discussed below, and the rest of the review will discuss the importance of Dectin-1–Syk–CARD9 signaling in orchestrating anti-mycobacterial immunity.

## Syk/CARD9-Coupled CLRs in TB Immunity

Dectin-2 recognizes mycobacterial mannosylated lipoarabinomannan (ManLAM) (5). This interaction results in the recruitment of immunoreceptor tyrosine-based activation motif (ITAM)-linked FcRγ, which links to Syk and CARD9, resulting in a cascade of downstream signaling and cellular activation (5, 6). Dectin-2 also induces production of anti- and pro-inflammatory cytokines IL-2, TNF, MIP-2, IL-6, and IL-10, in DCs stimulated with ManLAM and BCG. The interaction of this CLR with ManLAM has also been shown to induce T-cell responses (5). Dectin-2 deficiency results in increased pathological damage in mice infected with *M. avium* (5). Although Dectin-2 has been shown to recognize pathogenic Mtb strain, H37Rv, the *in vivo* protective role of this receptor against this strain is yet to be demonstrated.

Mincle interacts with mycobacteria *via* trehalose 6,6′ dimycolate (TDM) (7, 8), the most abundant glycolipid on the cell wall of the bacilli (9). Like Dectin-2, this receptor is also coupled to the adaptor molecule, FcRγ, which initiates Syk-mediated cellular responses (9, 10). Mincle has been shown to trigger pro-inflammatory cytokine production and nitric oxide (NO) in macrophages stimulated with TDM or its synthetic analog, trehalose 6,6-dibehenate (TDB). TDB also induces Mincle-driven adaptive Th1 and Th17 responses when used as an adjuvant to subunit vaccines in mice (7, 8, 11). Despite these contributions to protective responses, Mincle has been shown to be dispensable for the control of Mtb infection *in vivo* (12), although contradicting results have been reported (3, 4, 9).

Clecsf8 (MCL) is another FcRγ-coupled receptor that recognizes mycobacterial TDM (13). This CLR is known to positively regulate the expression of Mincle through a protein–protein complex interaction (14). Clecsf8-mediated cellular responses, which are dependent on the Syk/CARD9 complex, include phagocytosis, pro-inflammatory cytokine production, DC maturation, T-cell priming, and respiratory burst (14–16). Clecsf8-deficient mice are more susceptible to Mtb infection with increased lung bacillary loads, enhanced pathological damage with excessive neutrophilic infiltration, and early mortality (17). Clecsf8 polymorphisms are associated with TB susceptibility in humans (17).

Other Syk-coupled CLRs have been reported to recognize mycobacterial ligands. These have been reviewed elsewhere (4, 18), and they include DCAR, which recognizes glycolipds called PIMs (19), and SIGNR3, a DC-SIGN mouse homolog that recognizes ManLAM (20).

## Dectin-1 Structure and Function

**Figure 1:**
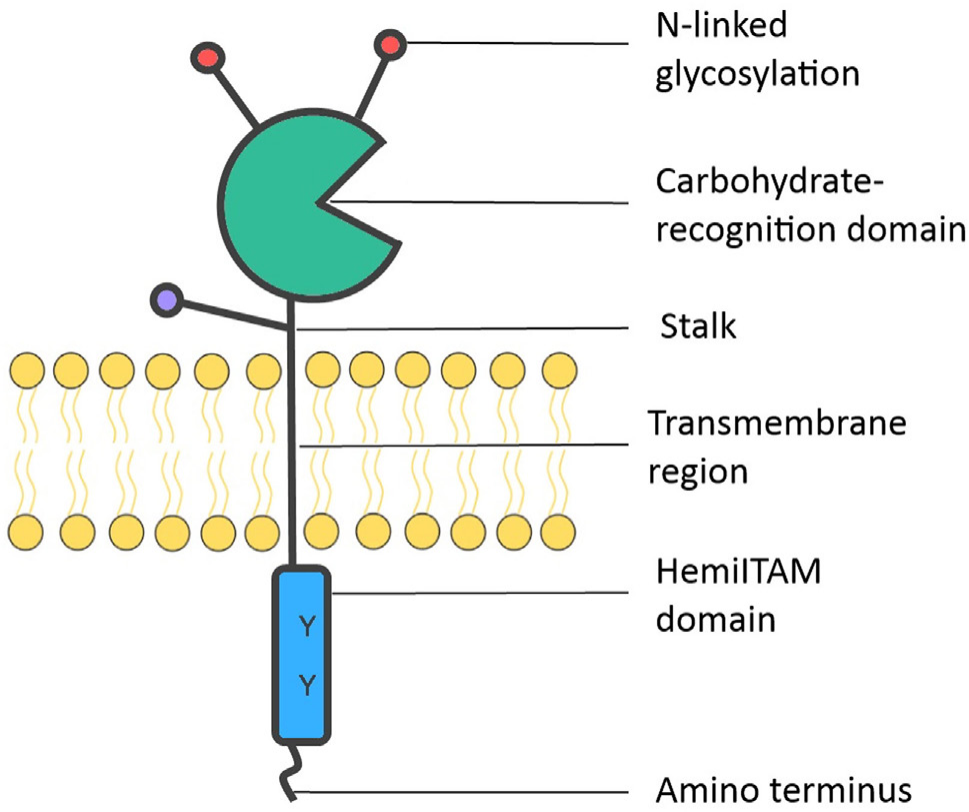
Dectin-1 structure. Dectin-1 consists of an extracellular C-type lectin domain, which is the carbohydrate-recognition domain (CRD) that binds ligands such as β-glucans. The CRD is attached by a stalk to a transmembrane region. Dectin-1 undergoes N-linked glycosylation on the CRD (mice) or stalk region (humans), shown in red and blue, respectively. The intracytoplasmic region comprises an immunoreceptor tyrosine-based activation motif- (ITAM)-like motif, or hemITAM, which initiates intracellular signaling.

Dectin-1 was initially identified by subtractive cDNA cloning, using mRNA extracted from murine DCs (21). This PRR is expressed on various myeloid cells, including macrophages and other mononuclear cells, as well as a subpopulation of T cells (22). Consistent with its role in pathogen surveillance (23), Dectin-1 is highly expressed by immune cells residing in the mucosa of the lung (22) and gut (24). Dectin-1 is a glycosylated transmembrane receptor (type II) composed of two functional domains (see Figure 1). An extracellular C-type lectin domain (CTLD) binds β-glucans, polysaccharides that occur as (1 → 3)-β-D-linked glucose polymers, mainly found on the surface of fungi, plants, and some bacteria (25). Dectin-1 may also bind ligands other than β-glucans (21), as evidenced by the observation that mycobacteria, which do not contain β-glucans on their cell wall, seem to interact with Dectin-1 through a yet to be identified ligand (26). The second functional domain is found on the intracellular tail region, which houses an ITAM-like motif, called hemITAM (23).

## Dectin-1 Signaling and Immune Responses

**Figure 2:**
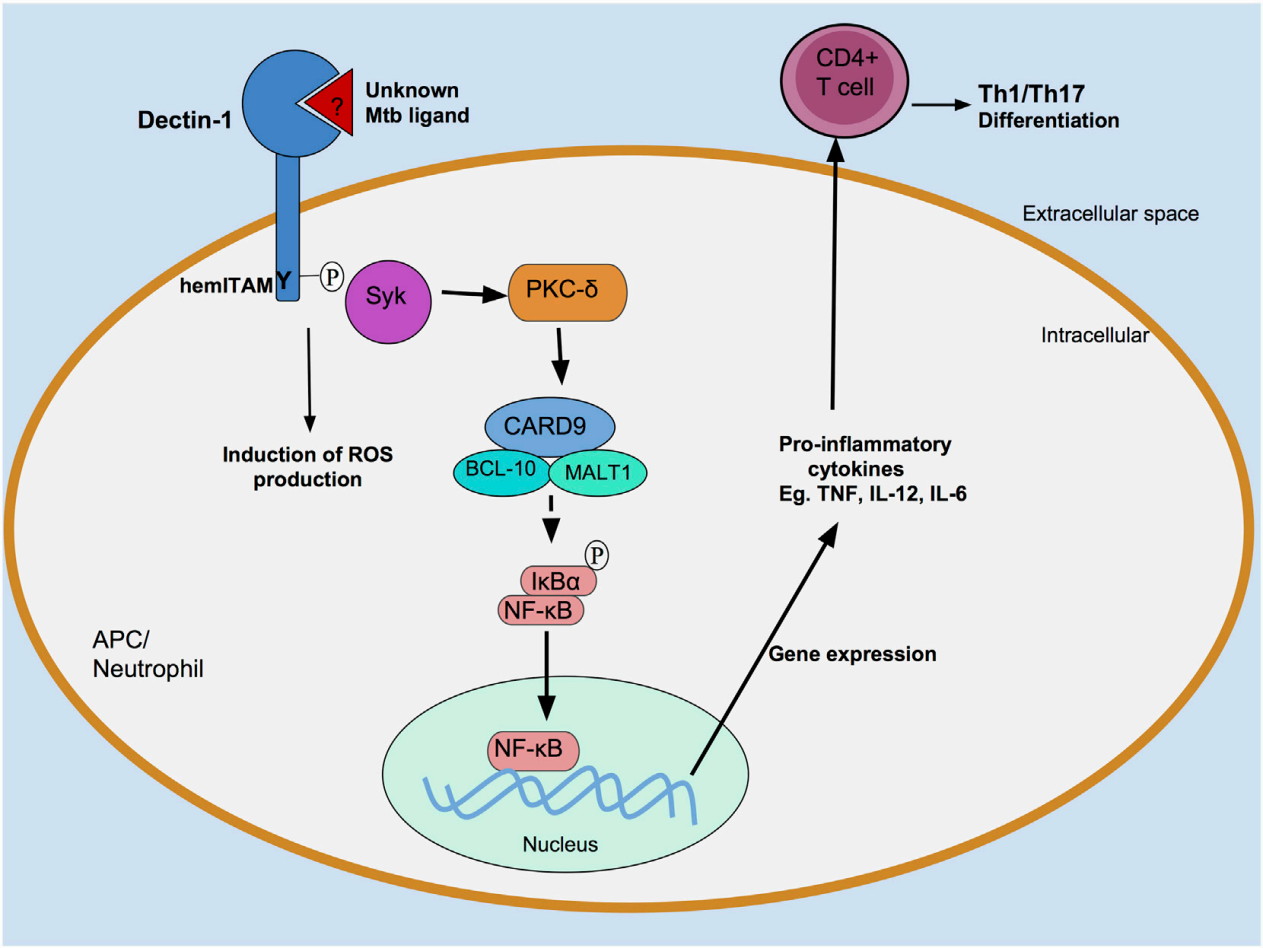
Recognition of *Mycobacterium tuberculosis* (Mtb) by Dectin-1. An unknown Mtb ligand is recognized by Dectin-1, which is then tyrosine phosphorylated at its hemITAM residue by Src kinases (data not shown). This creates a docking site for spleen tyrosine kinase (Syk). Syk associates with a caspase recruitment domain family member 9 (CARD9)/B-cell lymphoma 10 (BCL-10)/mucosa-associated lymphoid tissue lymphoma translocation protein 1 (MALT1) protein complex, resulting in activation of the transcription factor nuclear factor κB (NF-κB). Pro-inflammatory cytokine production follows, which induce an adaptive T cell response. Dectin-1-dependent signaling can also occur in neutrophils, where they induce reactive oxygen species (ROS) production.

Dectin-1 initiates intracellular signaling *via* its hemITAM motif (Figure 2). Following ligand engagement by the CTLD, Src kinases mediate the tyrosine phosphorylation of the hemITAM domain (23), creating a docking site for Syk, which initiates a series of intracellular signaling cascades resulting in activation of the transcription factor nuclear factor κB (NF-κB) (23, 27, 28). Dectin-1 can also drive non-canonical activation of NF-κB by utilizing the serine-threonine kinase Raf-1 (29).

By influencing gene expression, Dectin-1 activation can lead to numerous downstream cellular responses, including expression of cytokines such as TNF-α, IL-2, IL-10 and IL-12, and CXCL2 (28, 30, 31). Dectin-1-mediated activation can also induce phagocytosis (32) and respiratory burst (33). Furthermore, Dectin-1 signaling has been shown to orchestrate adaptive immunity. DCs activated by Dectin-1 agonists are capable of differentiating naïve CD4^+^ T cells to a T helper- (Th-)1 or Th17 phenotype both *in vitro* and *in vivo* (34). Dectin-1-activated DCs can also induce the maturation and proliferation of CD8^+^ T cells *in vitro*: the Dectin-1 agonist curdlan was found to act as an adjuvant for cytotoxic T lymphocyte cross-priming *in vivo*, which elicited potent responses capable of protecting mice from experimental tumor challenges (35). Thus, Dectin-1 signaling serves as a link that generates appropriate adaptive responses following immune recognition (36).

Dectin-1 does not appear to initiate protective responses in isolation, but acts synergistically with other receptors such as the TLRs. For instance, Dectin-1 stimulation was found to augment TLR-2-mediated production of cytokines in murine macrophages and DCs (30, 31). In addition, Ferwerda et al. showed that stimulation of human peripheral blood mononuclear cells (PBMCs) with a Dectin-1 ligand, as well as ligands for TLR-2 or -4, led to a synergistic increase in TNF-α production compared with Dectin-1 stimulation alone (37). Shin et al. extended this investigation to mycobacteria by infecting murine macrophages with *M. abscessus* (Mab), an environmental non-tuberculous *Mycobacterium* that can cause opportunistic infections in humans (38). The authors observed that Mab stimulation of macrophages initiated a physical colocalization between Dectin-1 and TLR-2 that was required for pro-inflammatory cytokine production (38). The mechanism underlying this apparent interaction remains to be elucidated (23, 39). In contrast to these findings, a study by Rothfuchs et al. showed that Dectin-1 inhibition significantly diminished the production of IL-12p40 by DCs lacking TLR-2 (40). This suggests that Dectin-1 signaling is not necessarily dependent on TLR-2. Notably, these conflicting studies described the effect of different microorganisms and stimuli on different cell types.

## Dectin-1 Recognition of Mycobacteria

Dectin-1 has been well characterized as a major fungal β-glucan receptor (41). Interestingly, Dectin-1 has also been shown to be involved in the innate immune recognition of mycobacteria (26), which do not contain β-glucans (40). Yadav and Schorey established this by infecting murine bone marrow-derived macrophages (BMDMs) with non-pathogenic *M. smegmatis*. They found that the production of TNF-α by BMDMs was decreased by approximately 60% in the presence of Dectin-1-blocking antibodies (26). Moreover, they showed that Dectin-1 was required for the production of IL-6, G-CSF, and RANTES by BMDMs (26). However, when the authors performed infection using more virulent mycobacterial strains, such as Mtb strain H37Rv, they observed a significant decrease in the production of TNF-α compared with non-pathogenic strains, and this minimal TNF-α production was not reliant on Dectin-1 (26). This suggested that the role of Dectin-1 in orchestrating immune responses to pathogenic mycobacteria is somewhat limited. In contrast to this finding, a later study by Rothfuchs et al. reported that Dectin-1 does interact with pathogenic mycobacteria (40). These authors demonstrated that murine splenic DCs (spDCs) infected with pathogenic Mtb produced significantly less IL-12p40 when treated with laminarin, a competitive inhibitor of Dectin-1 (40). Thus, Dectin-1 is involved in the recognition of the important human pathogen Mtb as well as less virulent mycobacteria. Rothfuchs et al. went on to show that pharmacological Syk inhibition reduced the capacity of spDCs to produce IL-12p40 upon Mtb exposure, suggesting that this response was Syk dependent (40). The authors also elegantly confirmed the presence of a Dectin-1 ligand on mycobacteria, by showing that a Dectin-1–Fc fusion protein (42) was capable of binding live *M. bovis* (40). These and other related studies established the role of Dectin-1-mediated responses in non-human innate immune cells. The role of this CLR in human cells was investigated more recently.

Although they are not traditionally considered to be immune cells, airway epithelial cells may serve an important function in mediating pulmonary immune responses. These cells express PRRs such as Dectin-1, albeit to a lesser extent than myeloid cells. Lee et al. reported that Mtb could induce Dectin-1 expression in human A549 airway epithelial cells in a TLR-2-dependent fashion (43), and such expression contributed to the production of reactive oxygen species (ROS), antimicrobial peptides, and pro-inflammatory cytokines by these cells (43). Another investigation into mycobacteria-induced Dectin-1 signaling in human cells was undertaken by Zenaro et al. These authors observed that Mtb infection induced the maturation of monocyte-derived DCs (MDCs), as well as the Dectin-1-dependent production of IL-1β, IL-6, IL-23, and TNF-α (44) by these cells. In addition, DCs activated with a Dectin-1 agonist stimulated naïve CD4^+^ T cells to secrete IFN-γ and IL-17 (44). Another study has demonstrated that simultaneous activation of neonatal MDCs with agonists of Dectin-1 and TLRs promote synergistic production of IL-12p70 (45). In human PBMCs stimulated with Mtb, Dectin-1 and TLR-4 are the main receptors driving IL-17A production (46). Interestingly, administration of Dectin-1 agonist, curdlan, together with a mycobacterial antigen TB10.4, induced Th1 and Th17 responses in neonatal mice infected with Mtb (45). A recent study by Bisiaux et al. investigated cell-specific activation and pro-inflammatory responses in human whole blood stimulated with BCG (47). Interestingly, Dectin-1/2 responses were predominantly activated in neutrophils when compared with monocytes and lymphocytes populations. This work also demonstrated that induction of ROS production by BCG was decreased by neutralization of Dectin-1/2 and TLR-2/4 in both neutrophils and monocytes (47). In agreement with these findings, a recent report has shown that Mtb can induce DC maturation by generating ROS production through Dectin-1/TLR-2 (48). These *in vitro* findings indicated that Dectin-1 signaling is involved in the stimulation and activation of neutrophils and antigen-presenting cells (APCs), which lead to adaptive anti-mycobacterial immune responses.

To explore the significance of Dectin-1 *in vivo*, Marakalala et al. investigated Dectin-1-deficient mice and wild-type (WT) controls infected with aerosolized Mtb (49). The kncokout mice had significantly and reproducibly decreased (~0.5 log) pulmonary bacillary burdens (49). However, both the Dectin-1-deficient and WT mice developed similar histological signs of pneumonia, suggesting that there was still profound inflammatory activation in Dectin-1-deficient mice despite their decreased bacterial burden. Indeed, Dectin-1 deficiency did not result in any significant and reproducible changes in the pulmonary cytokine expression profiles compared with WT controls (49). The authors also found that Dectin-1 deficiency did not significantly affect mouse survival 150 days of post-infection. Marakalala et al. concluded that Dectin-1 does not play a major role in immunity to Mtb *in vivo* and acknowledged the potential significance of the mechanisms underlying the decreased bacillary burden conferred by Dectin-1 deficiency.

## The Function of Syk in PRR Signaling and Immunity to Mtb

Spleen tyrosine kinase is an intracellular signal transducer that performs diverse biological functions, including innate immune recognition (50). Syk can be activated by associating with the phosphorylated ITAM or hemITAM motif of CLRs *via* one of its two SH2 domains. In the case of the Dectin-1/Syk pathway, Syk activates protein kinase C-delta (PKCδ) which mediates the phosphorylation of CARD9 (51). This enables CARD9 to associate with B-cell lymphoma 10 and the paracaspase mucosa-associated lymphoid tissue lymphoma translocation protein 1 (MALT1) to form a trimolecular structure capable of canonically activating NF-κB (52, 53). Such manipulation of gene expression allows Syk to exert its secondary messenger functions, triggering ROS production (33), accelerated phagocytosis (28), and the production of pro-inflammatory cytokines, such as IL-1β, IL-6, IL-12, and TNF-α (18). Mature IL-1β production requires further post-translational modification of pro-IL-1β by a caspase-1-containing oligomer called an inflammasome (50). The NLPR3 inflammasome is activated in a Syk-dependent mechanism by mycobacteria that express the virulence factor ESAT6, and such activation has been linked to macrophage necrosis (54). There is therefore mounting evidence that this signaling molecule may be relevant in immune responses to mycobacterial infections.

Spleen tyrosine kinase is involved in a multitude of biological functions, and therefore essential for normal survival in mice (55). This makes it difficult to determine the effect of Syk deficiency on mycobacterial immunity *in vivo*, for example, using a gene knockout study in mice. Better research tools are required to understand the role of Syk in mycobacterial infections *in vivo* and in humans.

## PKCδ and Immunity to Mtb

As already described, CLRs which activate Syk are linked to the CARD9–BCL10–MALT1 pathway. Strasser et al. showed that the linker molecule PKCδ is activated by Syk and mediates the phosphorylation of CARD9 (51). The role of this kinase in signal transduction prompted Parihar et al. to investigate the relevance of PKCδ function following Mtb infection (56). They observed that mice deficient in PKCδ (either through gene knockout or pharmacological inhibition) were less resistant to Mtb, displaying more severe lung pathology, excessive pro-inflammatory cytokine production, increased bacterial burdens, and increased mortality compared with their WT or untreated controls. Using gene expression profiles from Mtb-infected humans, the authors showed that PKCδ abundance was temporally associated with progression to TB disease. In addition, the authors analyzed lung specimens from TB patients and found that PKCδ was highly expressed in necrotic and cavitary granulomas (56). These data indicate that PKCδ is an important determinant of Mtb infection in humans and mice.

## CARD9 and Immunity to Mtb

CARD9 is an adaptor protein involved in the Dectin-1/Syk signaling cascade and is expressed in DCs and macrophages (52). The adaptor picks up on signals from multiple receptor classes, such as ITAM-based receptors (including CLRs) and TLRs (27). The role of CARD9 in Mtb immunity was evaluated by Dorhoi et al. using a mouse model of Mtb H37Rv infection (57). These authors demonstrated that CARD9-deficient mice had a reduced ability to control bacterial replication, developed severe lung pathology, and displayed increased mortality compared with their littermate controls (57). Infected CARD9-deficient mice developed acute pneumonia, and histological examination of the lungs revealed the presence of necrotic foci and an inflammatory infiltrate with profound neutrophil accumulation. Moreover, lung specimens from CARD9-deficient mice showed increased apoptotic cell death and secondary necrosis compared with WT controls (57). There is evidence to suggest that the mobilization of neutrophils from the bone marrow to peripheral tissues is dependent on the cytokines G-CSF and CXCL1 (58), and these cytokines were significantly elevated in the sera of CARD9-deficient mice compared with their littermate controls (57). The investigators also demonstrated that neutrophils deficient in CARD9 were unable to produce the regulatory cytokine IL-10 when challenged with Mtb, leading to deregulated pulmonary inflammation (57). These data led the authors to conclude that CARD9 is required for properly regulated innate immune cell activation during Mtb infection.

To further explore the mechanism of CARD9 function in TB, Dorhoi et al. infected APCs with Mtb H37Rv and found that deficiency in the CARD9 adaptor did not affect NO synthesis by the APCs nor did it affect the phagocytosis or destruction of Mtb following IFN-γ activation (57). However, CARD9-deficient BMDMs produced significantly less TNF-α, IL-1β, IL-6, IL-12, and CCL5 compared with WT controls (57). Given the reduced levels of IL-12 in their *in vitro* experiments, one might have expected to see impaired Th1 responses *in vivo* (as IL-12 contributes to Th-1 polarization). However, no such aberrations in T cell recruitment and activation were observed in the lungs of infected CARD9-deficient mice (57). Similarly, the authors did not observe any deficit in Th17 responses in CARD9-deficient mice (57), even though an earlier study demonstrated that the Syk/CARD9 pathway is required for developing Th17 responses to *Candida albicans* (34). Nevertheless, adequate T-cell responses were not able to overcome the pathology induced by defects in innate immune cell inflammatory activation. These results suggest an essential role for CARD9 in anti-mycobacterial immunity.

## The Syk/CARD9 Pathway in TB Vaccinology

To generate a protective immune response to Mtb, antigen-specific Th1 cells are required (59). In addition, the generation of Th17 cells secreting IL-17 has been shown to augment a protective host response by stimulating the influx of effector cells to the areas of infection (60). An effective vaccine to Mtb would probably need to activate APCs in such a way that they “instruct” T cell differentiation to Th1 and Th17 phenotypes. One novel vaccination strategy involves using recombinant Mtb antigens as subunit vaccines; however, these vaccines have not been successful on their own, perhaps because in themselves they do not activate APCs. This necessitates the use of vaccine adjuvants capable of triggering helpful innate cellular responses to guide adaptive immunity. Potential adjuvants include the mycobacterial PAMPs TDM and its synthetic analog TDB, which induce protective Th1 and Th17 immunity by activating APCs *via* the Syk–CARD9–BCL10–MALT1 pathway (11). The major PRRs that bind TDM are Mincle (7, 8) and Clecsf8 (13, 17), suggesting that signaling initiated by either one or both of these CLRs *via* the Syk/CARD9 pathway may be essential for protective immunity to Mtb.

## Conclusion and Future Directions

An important step toward a deeper understanding of Dectin-1–Syk–CARD9 signaling will be identifying the mycobacterial PAMP recognized by Dectin-1. This would help elucidate how Mtb interacts with human innate immune cells, and how this contributes to or lessens pathology. β-Glucans are currently the only known Dectin-1 ligand, yet Dectin-1 recognizes mycobacteria, which do not express β-glucans. The nature of the ligand will therefore broaden our knowledge of Dectin-1 PAMP recognition.

Dectin-1 itself may not be essential for Mtb immunity, but some of the downstream signaling molecules such as CARD9 evidently are. The effect of other signaling molecules, like Syk and PKCδ, in Mtb responses remains to be fully elucidated. In addition, future studies should look at how CLRs that utilize this signaling pathway interact, perhaps by performing gene knockouts of multiple receptors in mice. Although Dectin-1 appears to play a minor role in *in vivo* Mtb immunity, components of Dectin-1/Syk signaling can induce protective downstream host responses, and this pathway remains a potential target for vaccine adjuvants.

## Author Contributions

MW wrote the manuscript. All authors planned the manuscript content, analyzed the literature, wrote parts of, and edited the manuscript.

## Conflict of Interest Statement

The authors declare that the research was conducted in the absence of any commercial or financial relationships that could be construed as a potential conflict of interest.
